# The clinical application of beta-lactam antibiotic therapeutic drug monitoring in the critical care setting

**DOI:** 10.1093/jac/dkad223

**Published:** 2023-07-19

**Authors:** Rekha Pai Mangalore, Trisha N Peel, Andrew A Udy, Anton Y Peleg

**Affiliations:** Department of Infectious Diseases, Alfred Health, 55 Commercial Road, Melbourne, Victoria 3004, Australia; Department of Infectious Diseases, Central Clinical School, Monash University, 99 Commercial Road, Melbourne, Victoria 3004, Australia; Department of Infectious Diseases, Alfred Health, 55 Commercial Road, Melbourne, Victoria 3004, Australia; Department of Infectious Diseases, Central Clinical School, Monash University, 99 Commercial Road, Melbourne, Victoria 3004, Australia; Department of Intensive Care and Hyperbaric Medicine, Alfred Health, 55 Commercial Road, Melbourne, Victoria 3004, Australia; Australian and New Zealand Intensive Care Research Centre (ANZIC-RC), School of Public Health and Preventive Medicine, 553 St Kilda Road, Melbourne, Victoria 3004, Australia; Department of Infectious Diseases, Alfred Health, 55 Commercial Road, Melbourne, Victoria 3004, Australia; Department of Infectious Diseases, Central Clinical School, Monash University, 99 Commercial Road, Melbourne, Victoria 3004, Australia; Biomedicine Discovery Institute, Department of Microbiology, Monash University, Clayton, Victoria 3800, Australia

## Abstract

Critically ill patients have increased variability in beta-lactam antibiotic (beta-lactam) exposure due to alterations in their volume of distribution and elimination. Therapeutic drug monitoring (TDM) of beta-lactams, as a dose optimization and individualization tool, has been recommended to overcome this variability in exposure. Despite its potential benefit, only a few centres worldwide perform beta-lactam TDM. An important reason for the low uptake is that the evidence for clinical benefits of beta-lactam TDM is not well established. TDM also requires the availability of specific infrastructure, knowledge and expertise. Observational studies and systematic reviews have demonstrated that TDM leads to an improvement in achieving target concentrations, a reduction in potentially toxic concentrations and improvement of clinical and microbiological outcomes. However, a small number of randomized controlled trials have not shown a mortality benefit. Opportunities for improved study design are apparent, as existing studies are limited by their inclusion of heterogeneous patient populations, including patients that may not even have infection, small sample size, variability in the types of beta-lactams included, infections caused by highly susceptible bacteria, and varied sampling, analytical and dosing algorithm methods. Here we review the fundamentals of beta-lactam TDM in critically ill patients, the existing clinical evidence and the practical aspects involved in beta-lactam TDM implementation.

## Introduction

Beta-lactam antibiotics (beta-lactams) are defined as ‘time-dependent’ antibiotics. Their bactericidal activity is dependent on the pharmacokinetic/pharmacodynamic (PK/PD) index, *fT* > MIC: the proportion of time (*T*) their unbound concentration (*f*) remains above the MIC of the infecting bacterium.^1^ The evidence for this PK/PD index stems from *in vitro* and animal model studies and, together with PK studies conducted in healthy human volunteers, forms the basis of dosing recommendations for beta-lactams.^2–5^ Patients who are severely unwell have a range of physiological changes that can greatly affect the PK of beta-lactams.^6^ Large fluid shifts, hypovolaemia, hypoalbuminaemia, hypotension and organ dysfunction affect the volume of distribution and elimination of beta-lactams.^7–9^ These alterations often occur concurrently and can lead to either sub-therapeutic concentrations, which may affect treatment response and increase the risk of resistance, or supra-therapeutic concentrations, which may cause toxicity.^3,7,10^ Moreover, clinical trials assessing the efficacy of beta-lactams often exclude patients with very high Acute Physiology and Chronic Health Evaluation Scores of ≥30, refractory shock and renal replacement therapy, further supporting the idea that beta-lactam dosing recommendations are not tailored to critically unwell patients most at risk of variability in exposure.^11–13^

Therapeutic drug monitoring (TDM) involves the measurement of a drug concentration to help inform optimized dosing, with the goal of improving patient outcomes and minimizing toxicity.^5^ TDM guided dose adaptation can address individual patient-level PK variability and improve the attainment of therapeutic concentrations.^14,15^ For aminoglycosides, TDM has led to reduced toxicity and shorter hospital length of stay.^16^ The 2021 surviving sepsis guidelines recommended antimicrobial dose optimization based on PK/PD principles and TDM.^17^ Given that beta-lactams are one of the most common antibiotics prescribed in sepsis, beta-lactam TDM should be a key priority.^5,7^ Thus far, only a few centres worldwide perform beta-lactam TDM, with reasons for the low uptake including a lack of prospective clinical trials demonstrating benefits, the reassuring safety profile of beta-lactams, the infrastructure and resources required from a laboratory and health service perspective, the lack of availability of dosing guidance and the lack of pharmacoeconomic data on cost-effectiveness.^18–21^ This narrative review will discuss the fundamentals of beta-lactam TDM in critically ill patients, the existing clinical evidence and the practical aspects involved in beta-lactam TDM implementation.

## Pharmacokinetic/pharmacodynamic considerations of beta-lactams in critically ill patients

Beta-lactams are hydrophilic and primarily eliminated by the kidneys, making them particularly subject to changes in volume of distribution and elimination.^22^ The physiological changes that occur in patients who are critically ill can have significant effects on beta-lactam pharmacokinetics.^22^ Fluid shifts between organs and tissue spaces, hypovolaemia and hypotension, fluid resuscitation, hypoalbuminaemia, organ supports such as extracorporeal membrane oxygenation (ECMO) and renal replacement therapy, and organ dysfunction, all affect the volume of distribution and consequently plasma concentrations of beta-lactams.^7–9^ Changes in renal function seen in critically ill patients such as impaired or augmented (CL(Cr) ≥130 mL/min) renal clearance can lead to decreased or increased elimination of these drugs, respectively.^23,24^ Studies evaluating the clinical implications of some of these physiological changes are described next and readers are also referred to several reviews for a detailed outline of the PK variability of beta-lactams in critically ill patients.^25–28^

*In vitro* and *in vivo* animal model studies have demonstrated that the efficacy of beta-lactams is achieved if the *fT* > MIC is maintained for 40% of the dosing interval for carbapenems, 50% for penicillins and monobactams and 50%–70% for cephalosporins (Table 1).^1,2,37^ The bactericidal effect has been shown to be maximal at 4–5×MIC and plateaus at concentrations above this threshold.^4^ Much debate still exists on the optimal concentration targets for beta-lactams in clinical care.^38^ Higher PK/PD targets of 100%*fT* > MIC and 100%*fT* > 4×MIC have been called for in critically ill patients, and a recent position paper endorsed by European working groups have recommended aiming for 100%*fT* > MIC, with minimum steady-state concentration monitoring for intermittent infusions (*C*_min_, sample obtained prior to next dose) and steady-state concentrations for continuous infusions (*C*_ss_, sample obtained at any time during infusion).^14,39^

**Table 1.** Pharmacokinetic/pharmacodynamic targets for beta-lactam antibiotics for efficacy and toxicity

Beta-lactam class	PK/PD target (efficacy)^a^	PK/PD threshold for toxicity^b^
Penicillin	≥50%*fT *> MIC	Variable depending on organ involved and type of beta-lactam. *f*T > 6–10×MIC is considered toxic.
Cephalosporin	40%–70%*fT *> MIC	
Carbapenem	40%*fT* > MIC	
Monobactam	50%*fT* > MIC	
Beta-lactam (all classes) threshold in critically ill	100%*fT* > MIC or 100%*fT* > 4×MIC	Neurotoxicity PIP*: C*_min_ > 361.4 mg/L^10^ MEM: *C*_min_ > 64.2 mg/L^10^ FLX: *C*_min_ > 125.1 mg/L^10^ FEP: *C*_min_ > 20 mg/L^29-35^ FEP: *C*_ss_ > 60 mg/L^36^ Nephrotoxicity PIP: *C*_min_ > 452.65 mg/L^10^ MEM: *C*_min_ > 44.45 mg/L^10^

Targets for efficacy based on animal studies, *in vitro* studies and some clinical studies; FLX, flucloxacillin.

No clear toxicity threshold has been identified, threshold values derived from observational pharmacokinetic and retrospective studies.

## Clinical studies evaluating beta-lactam PK/PD in critically ill patients

Observational clinical studies have thus far explored the impact of various beta-lactam levels on clinical and/or microbiological outcomes, and provide support for the recommendation of achieving greater time above MIC for beta-lactam concentrations.^40–42^ The Defining Antibiotic Levels in Intensive Care (DALI) study by Roberts *et al.* was a multinational, point prevalence, beta-lactam PK study of eight different beta-lactams, which included 248 patients with infections, and found that patients who did not achieve 50%*fT* > MIC were 32% less likely to have positive clinical outcomes.^14^ In the multivariate model they observed higher PK/PD index of 100%*fT* > MIC was associated with a 56% greater probability of a positive clinical outcome.^14^ These data were supported by Ariano *et al.* who studied 60 critically ill febrile neutropenic patients and showed an 80% clinical response in those treated with meropenem who achieved 75%*fT* > MIC, whereas, an average exposure of 59%*fT* > MIC was observed in patients with poor response.^40^ Moreover, Al-Shaer *et al.* conducted a study of 206 critically ill patients with a range of infections using actual MICs to evaluate exposure, and showed that the odds of microbial eradication and clinical cure were more than doubled (aOR 2.56, 1.01–6.51) and tripled (aOR 3.00, 1.11–8.12), respectively, in those who achieved 100%*fT* > MIC.^43^ Very little clinical data exist to support higher concentrations at multiples above the MIC. A small prospective open-label study of patients treated with cefepime (*n* = 36; 20 patients with Gram-negative infections with MICs determined by Etest) showed that cefepime serum concentrations of 83% and 95% *fT* > 4.3×MIC were associated with 80% and 90% probability of microbiologic success, respectively.^44^ The study by Al-Shaer *et al.* also showed that exposure of 100%*fT* > 4×MIC significantly reduced the emergence of new resistance by 79% (aOR 0.21. 0.07–0.62).^43,45^ Further clinical studies are still required to determine whether higher concentrations of beta-lactams as a multiple of MIC are beneficial for critically ill patients.

Similar to 100%*fT* above a multiple of MIC, trough concentration (*C*_min_) to MIC ratio (*C*_min_/MIC) has also been proposed as a drug concentration target for efficacy. *C*_min_/MIC ratios of >1.3–5 have been associated with greater clinical or microbiological success in PK studies of various beta-lactams.^41,46^ Using population models, Aitken *et al.* and Li *et al.* demonstrated that a *C*_min_/MIC ratio >5 for meropenem, which equates to 100%*fT* > 5×MIC, and *C*_min_/MIC ratio >2.1 for cefepime, were associated with improved clinical and microbiological outcomes, and reduced clinical failure, respectively, in the treatment of lower respiratory tract infections.^33,47^ These data were supported by Wong *et al.* who evaluated data from 98 patients treated with meropenem (*n* = 46), piperacillin/tazobactam (*n* = 36) and ceftazidime (*n* = 10) for Gram-negative bloodstream infections. Using CART analysis, they observed that the ratio target of *fC*_min_/MIC >1.3 was significantly associated with improved outcomes.^46^ A more recent study in ICU patients with Gram-negative bacteraemia (*n* = 44) applied this efficacy target when using a continuous infusion (meropenem) and showed that a steady-state concentration (*C*_ss_) to MIC (*C*_ss_/MIC) ratio of ≥4.63 was associated with improved clinical outcomes.^48^*C*_min_/MIC ratios have also been shown to be associated with suppression of antimicrobial resistance in hollow-fibre infection models, including ≥3.4 for intermittent bolus infusions and ≥10 for prolonged infusions of piperacillin/tazobactam and ≥3.8 for cefepime, ceftazidime and meropenem.^49,50^ However, clinical data are lacking to support these *in vitro* model findings and a systematic review by Sumi *et al.* concluded that no recommendations on PK/PD targets for Gram-negative resistance suppression could be made based on available data.^51^

Observational studies have illustrated the challenges of beta-lactam PK variability on outcomes in severely unwell patients. Roberts *et al.* studied a cohort of 24 critically ill patients undergoing continuous renal replacement therapy and showed variability in trough concentrations of 6.7-fold for meropenem and 3.8-fold for piperacillin.^52^ In a follow-up study of 381 patients on renal replacement therapy, >25% did not achieve high concentrations, defined as free beta-lactam trough concentrations four times above MIC, and 4% failed to reach lower target concentrations, defined as free trough concentration over MIC for piperacillin and meropenem.^24^ Hypoalbuminaemia is another common finding in critical illness with a reported incidence of more than 40%–50% and is particularly important for certain beta-lactams that are highly protein bound (>80%) such as ceftriaxone, flucloxacillin and ertapenem.^53,54^ The resultant unbound concentrations can be unpredictable, and much of the unbound drug can escape into extra-vascular third spaces or undergo rapid renal clearance.^54,55^ It is recommended that free drug concentrations be measured for TDM of highly protein-bound beta-lactams and some authors recommend administration of higher initial loading doses in cases of severe hypoalbuminaemia.^55,56^ Patients with burns are also at higher risk of poor target attainment. In a prospective cohort study of 50 patients admitted with burns and treated for infection, 60% did not achieve beta-lactam target concentrations.^57^ In general, critically ill patients often have multiple drivers of altered pharmacokinetics and it is in these patient populations where TDM guided dose adjustment may have the greatest benefit.

The other major benefit of TDM, and what has made aminoglycoside and vancomycin TDM standard of care, is to reduce the risk of toxicity. Beta-lactams have a wide therapeutic index and are considered to be safe even at high doses, however, neurotoxicity, hepatotoxicity, nephrotoxicity and bone marrow suppression (cytopaenia) have all been described in patients on treatment with beta-lactams.^29,58^ The exact PK/PD threshold for toxicity is uncertain and varies with different beta-lactams.^10,29^ Neurotoxicity has been reported due to cefepime accumulation in critically ill patients with renal impairment.^30–32^ A systematic review of 37 studies by Payne *et al.* concluded that there may be a causal relationship between cefepime and neurotoxicity, particularly with larger doses of cefepime in renal impairment but can also occur in patients on appropriate doses without renal impairment.^33^ The authors noted median cefepime serum and CSF concentrations to be 45 mg/L in 21 patients and 13 mg/L in four patients, however, no conclusion could be made between concentrations and neurotoxicity.^33^ Beumier *et al.* (*n* = 199) identified that elevated *C*_min_/MIC ratios (risk greatest with *C*_min_/MIC ≥ 8) for meropenem, piperacillin, ceftazidime and cefepime correlated with neurotoxicity, and Huwyler *et al.* found a 5-fold increased risk of neurotoxicity in patients with cefepime *C*_min _> 20 mg/L.^34,35^ Toxicity thresholds for continuous infusions of cefepime have also been assessed, with a cefepime steady-state concentration (*C*_ss_) of >63.2 mg associated with neurotoxicity in a retrospective cohort study (*n* = 98).^36^ Imaini *et al.* (*n* = 378) identified threshold beta-lactam trough concentrations at which there was a 50% risk of emergent neurotoxicity and nephrotoxicity; they found no association with hepatotoxicity.^10^ Despite beta-lactams being considered safe, high doses are often used in intensive care setting and risk of toxicity are greater in this patient population. Further research on beta-lactam toxicity thresholds when using higher doses in critically ill patients is still warranted.

## Clinical studies evaluating the role of beta-lactam therapeutic drug monitoring in clinical care

TDM guided dose adaptation builds on the PK/PD principles of pre-clinical and clinical studies, and translates these findings to the bedside with the goal of improving patient outcomes. Clinical data on beta-lactam dose adaptation using TDM predominantly comes from a small number (*n* = 6) of randomized controlled trials (RCTs) and cohort studies (most of which are retrospective).^59–71^ Two of the most recent RCTs by Ewoldt *et al.* (*n* = 388) and Roggeveen *et al.* (*n* = 252) assessed the impact of beta-lactam dose individualization using TDM and model informed precision dosing (MIPD) software on clinical outcomes of critically ill patients compared to standard dosing.^63,64^ Neither study showed a statistically significant impact on patient outcomes, length of stay or target attainment. The trial by Roggeveen *et al.* allowed for enrolment of patients who had been on antibiotics for a longer period of time leading to a delay in dose optimization, which may have underestimated the effect of dose optimization.^64^ There were similar delays (>36 hours) to dose optimization in the trial by Ewoldt *et al*.^63^ Another recent multicentre RCT by Hagel *et al*. involving 249 patients (124 in the control group; 125 in TDM group; 13 sites) receiving piperacillin via a continuous infusion, assessed the effect of TDM guided dose optimization on organ dysfunction.^62^ They found no statistically significant change in the sequential organ failure score (ΔSOFA) or 28-day mortality between the two groups, however, there was improved target attainment in the TDM group when compared with the non-TDM group.^62^ Moreover, patients were allowed to receive concomitant antibiotic therapy, which may have influenced outcomes.^62^ In a small RCT of 38 patients with burns, TDM resulted in greater target attainment [aOR, 2.34 (95% CI, 1.17, 4.81)] but no difference in clinical resolution of infection, when compared with the no-TDM, standard of care group.^59^ This study did not report on renal clearance, however, patients with burns are at increased risk of augmented renal clearane that can impact beta-lactam concentrations.^72^ Two other small RCTs similarly showed improved target attainment.^60,61^ All three RCTs were not powered to detect an improvement in clinical outcomes.^60–63^

As a consequence of multiple RCTs with small sample sizes, several systematic reviews and meta-analyses have been performed, and have shown that a TDM guided approach in critically ill patients provided an 85% higher target attainment, 17% and 14% higher clinical and microbiological cure, respectively, and a 21% reduction in treatment failure.^73^ While no mortality benefit was demonstrated, this systematic review highlighted the utility of TDM in providing a tailored approach to beta-lactam dosing in critically ill patients who are at risk of therapeutic failure.^73^ Another recent systematic review and meta-analysis analysed the overall impact of antimicrobial TDM (beta-lactams, glycopeptide, voriconazole) and similarly found improvement in target attainment, treatment failure and reduction in nephrotoxicity but no impact on mortality.^74^

The lack of evidence on survival benefit has been a limitation to the uptake of beta-lactam TDM when compared with other antimicrobial TDM.^20^ However, there are important reasons why mortality benefits may have not been demonstrated in the clinical studies published thus far. First, patients who are critically ill and admitted to ICUs often receive beta-lactam antibiotics but may never culture a causative organism or have an infection at all yet are still included in beta-lactam trials. By including these patients, the results are more likely to support the null hypothesis that there is no difference between the intervention arms. Second, for most study participants who do have a causative bacteria cultured, the MICs of the beta-lactam antibiotics are most often low. For example, in the study by Hagel *et al.*, nearly 80% of the pathogens isolated from 166 patients had MICs to piperacillin ≤4 mg/L.^62^ In the context of very low MICs, the likelihood is that most dosing would be adequate, therefore underestimating the clinical impact of beta-lactam TDM. Furthermore, and as represented by the Hagel *et al.* study, the control group may already be receiving high dose or extended/continuous infusion beta-lactams that would already improve PK/PD target attainment.^62^ Third, as described, targets for efficacy rely on the MIC of the infecting pathogen, however many studies assessing target attainment use ‘worst case MICs’ i.e. epidemiological cut-off values (ECOFF) or the EUCAST clinical breakpoints (http://www.eucast.org/clinical_breakpoints; https://mic.eucast.org/search/) rather than actual MICs or local antibiograms. Using higher MIC values can underestimate the PK/PD target achieved.^37^ Fourth, TDM guided dose optimization may have no benefit in patients that are only mildly unwell or those that are severely unwell whereby no variation in treatment would change their outcome. There is probably a sweet spot in the middle that would benefit most from TDM guided dose optimization, however, there are challenges in defining that group and tailoring study inclusion. Other limitations with the existing literature that provide important insights into future study design include a lack of blinding of the study investigators, which may introduce bias in the outcome analysis, logistical delays with the intervention as was seen in the evaluation of MIPD tools, and clearly defining outcome definitions that are consistent and well recognized definitions to improve the generalisability of the studies.^63,64,75^ These common limitations should be strongly considered when designing future studies that assess the impact of beta-lactam TDM in critically ill patients.

Given the results from the systematic reviews and observational studies, plus the limitations of the existing RCTs, we support the use of beta-lactam TDM guided dose adaptation in patients at risk of sub-therapeutic concentrations due to PK variability (e.g. augmented renal clearance, RRT and major burns) and those with PD characteristics that predispose them to poor clinical outcomes. These include patients with difficult-to-treat infections such as prosthetic device infections, deep-seated cardiac and CNS infections, and infections with multidrug-resistant organisms with high MICs in patients for whom high doses or prolonged courses of beta-lactam therapy are often required.^70,72,76–80^

## Implementation considerations for beta-lactam TDM

**Figure 1.**
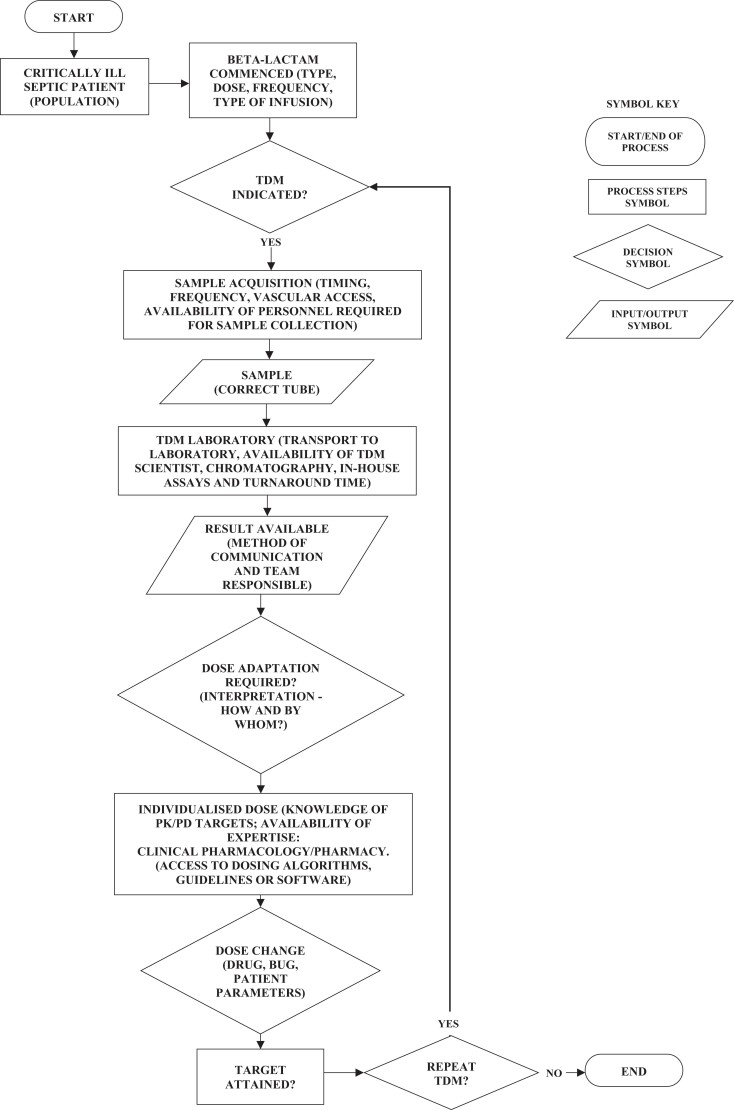
Process flowchart of TDM.

Successful implementation of beta-lactam TDM into a hospital system requires a comprehensive workflow (Figure 1). Most of the components of this workflow are established for TDM of other antimicrobials such as aminoglycosides and vancomycin, but these need to be adapted for beta-lactam specific purposes. In the following sections of the review, we will discuss some of the key practical aspects that need to be considered when establishing a beta-lactam TDM service.

### Sampling, access to assays and timing of sampling

As discussed previously and shown in Table 2, there are certain patient populations that would benefit most from beta-lactam TDM and would provide a starting point for eligibility. To be worthwhile, TDM should be performed if prolonged beta-lactam therapy (e.g. at least >48 hours) is expected. For maximum clinical utility, having a laboratory that can run samples 7 days a week would be ideal but is often not possible.^20^ The need for analytical systems and dedicated equipment (e.g. LC-MS/MS), and expertise in laboratory staff, are required for the successful application of beta-lactam TDM.^81^ Typically, beta-lactam assays are developed in-house, as standardized commercial assays are not available.^81^ Several assay methods exist and these vary in their ease of use, turnaround times and sample preparation.^81^ Liquid chromatography (e.g. LC-tandem MS, UPLC) machines require trained personnel, standardization and quality assurance practices. They also have high acquisition and maintenance costs and are mostly available in central laboratories of referral hospitals.^82^ Many of the clinical studies reporting a beta-lactam TDM service were only available between Monday and Friday, and varied from 2–4 days per week, which may limit their overall clinical utility.

**Table 2.** Indications for beta-lactam TDM

Indications
Critically ill with suspected or proven sepsis
Augmented or reduced renal clearance
ECMO and CRRT
Special populations: Immunocompromised, severe burns, extremes in body size, i.e. very low BMI/cachexia or morbid obesity^a^
Deep-seated infections^a^, e.g. CNS, bone and joint, heart valve, pacemaker, vascular graft
Incomplete source control or high inoculum infections^a^
Infections with less susceptible organisms
Suspected toxicity, i.e. cefepime, imipenem and meropenem

Limited data, authors’ recommendations; CRRT, continuous renal replacement therapy.

The timing of sample collection is crucial in TDM interpretation and dose optimization particularly for intermittent infusions.^83,84^ Most of the published beta-lactam TDM studies report sample collection at steady state after four doses for intermittent bolus infusion or four half-lives for continuous infusions.^60–62^ Trough (*C*_min_) samples are most often collected for intermittent bolus and extended infusions (over 3 hours). Ideally two samples (mid-point and trough) should be collected to account for assay limitations, however, trough samples may be the most practical and convenient to obtain in busy clinical settings.^15^ The availability of personnel for repeated timed blood draws is an important consideration. For continuous infusions samples can be obtained at any time once steady state is reached.^15^

The next important consideration includes the identification of the most appropriate team for interpretation of the TDM results and subsequent dose adaptation advice, and the best method of communicating the results to the clinical teams.^85^ Using existing pathways of conveying laboratory results appears to be the most effective and convenient, and having a pharmacy-led approach, which has been shown to be successful in the case of vancomycin and aminoglycoside TDM.^86^ Developing pathways to expand the scope of pharmacists would be a useful step when considering implementation.^86^

### Minimum inhibitory concentrations and dose adaptation

The primary goal of dose adaptation is to achieve a PK/PD target, which is a relationship between the serum levels of an antimicrobial and the MIC of that antimicrobial for a given causative organism. The MIC is a measurement of the antimicrobial activity against a standard inoculum of a bacterial strain under standardized laboratory conditions.^87^ The value assigned to the MIC depends on the antibiotic concentration at which visible growth is observed.^87^ In clinical practice, the MIC is measured using semi-automated machines (e.g. VITEK) and is subject to variability.^87^ For antibiotic dose adaptation, where target attainment involves a relationship between the antibiotic level and the MIC, experts have argued against the use of a single ‘snapshot’ MIC. Mouton *et al.* have discussed the limitations of the MIC and have proposed the use of the ECOFF, which is the highest MIC of an antibiotic from a group of isolates that do not show phenotypic resistance (wild-type strains).^87,88^ They have proposed two scenarios to interpret the MIC for target attainment: in the first scenario, the ECOFF value is used if the measured MIC is below or equal to the ECOFF, and in the second scenario, if the MIC is greater than the ECOFF, they suggest using the MIC plus a 2-fold dilution.^87^ Using the ECOFF value is useful for dose optimization with empiric therapy, when antimicrobial susceptibility data is not available, or in situations where the microbiology laboratory susceptibility report does not include MIC values. This latter situation is common, which makes the second scenario suggested by Mouton *et al*. challenging to implement as MICs are often not reported.^87^ An alternate option if MIC values are not routinely reported is to use local antibiogram data. The impact of dose adjustment to target for maximum *T* > ‘worst case MIC/highest MIC’ values on clinical and toxicological outcomes warrants further study.^89^

Thus far, the dose adaptation algorithms reported by most studies has been based on expert opinion, with little evaluation of their success.^89^ The most common approach has been the ‘rule of three’, which involves increasing or decreasing the dose, the frequency and/or the dosing interval.^90^ In most cases, repeat sampling of patients after dose adjustment has been uncommonly performed or reported on. In studies where repeat sampling was reported, a significant proportion (up to 40%) did not achieve desired targets despite dose adaptation.^5,35,38,69^ Newer dose optimization software for beta-lactams that integrates population PK models, Bayesian forecasting (MIPD) and electronic health record linkage, are becoming available and will provide a more precise approach to dose optimization.^90–94^ Software for dose adaptation and MIPD has been reviewed comprehensively elsewhere, but it is relevant to note that validation of these programs in the clinical context has not been performed.^75,90,95^

### Assessing effectiveness

Successful implementation of beta-lactam TDM needs continual evaluation of the effectiveness of the process and the clinical benefits.^96^ Defined clinical outcomes such as clinical cure or microbiological eradication from sterile samples or improvement in organ function such as time to vasopressor or ventilator-free days are evaluable endpoints. Routinely collected data such as length of stay, antibiotic doses and consumption can also be used in evaluation.^87,96–98^ In their systematic review, McAleenan *et al.* evaluated the methodological features of clinical PK/PD studies of antibacterial and antifungal agents and suggested guidelines for conducting such studies in the clinical context.^99^ Key recommendations included a robust sample size and statistical analysis and reporting; choice of a homogeneous population and appropriate population PK models; use of standardized susceptibility testing and validation with another laboratory; use of clear, standardized, patient relevant outcomes and evaluation of PK/PD indices for efficacy achieved in clinical settings relative to pre-clinical PK/PD studies.^99^

Another important aspect of successful continuation of a TDM programme is the evaluation of cost-effectiveness. Antibiotic TDM cost-effectiveness has been studied in the context of aminoglycosides.^16,100^ Costs of set-up, ongoing maintenance, technical support and training need consideration. In their study of ventilator-associated pneumonia, Duszynska *et al.* (*n* = 16, continuous infusion of piperacillin) showed a 37% reduction in the total dose with TDM guided continuous infusions of piperacillin versus intermittent bolus dosing, demonstrating a cost reduction of €15/patient/day or €105 for 7 days of treatment.^101^ Aldaz *et al.* noted a 66.2% reduction in daily meropenem dose in patients that underwent monitoring.^69^ It can be hypothesized that achieving effective targets early may result in earlier control of sepsis, shorter ICU and hospital length of stay, lower antibiotic daily doses, shorter antibiotic duration and an overall lower risk of colonization with or emergence of antibiotic-resistant organisms, all leading to reduced costs.^100^ The pharmacoeconomic data proving cost-effectiveness of TDM to date are limited and conflicting, with a *post hoc* analysis of the EXPAT study showing no difference in cost-effectiveness in patients that attained target concentrations; and RCT data from the Ewoldt *et al*. study showing higher costs in the TDM/dose optimization group compared with standard of care group.^63,102^ A dedicated approach to cost–benefit analysis in future research is needed is needed to establish the economic value of beta-lactam TDM.^103^

### Stakeholder engagement

Finally, the successful operation of a TDM program requires prescribers to change their usual practice.^104^ Individualized dosing requires use of doses higher or lower than the standard recommended doses or may involve the use of prolonged or continuous infusions and loading dose administration.^89,105,106^ Physicians may not find this change acceptable. Continuous infusions require dedicated vascular access placing additional burden on nursing workload.^105,107^ They also need knowledge of stability of various beta-lactams, for example, meropenem remains stable for about 4 h at a temperature of 25°C and needs to be cooled to 4°C to maintain stability for 24 hours.^108,109^ Schoenenberger-Arnaiz *et al.* found that TDM guided dose recommendations were not accepted in more than one-third of cases.^110^ They hypothesized that the lack of knowledge and low frequency of sampling (sample sent on third or fourth day for most patients during the course of antibiotic treatment) may be key reasons underlying the reluctance to accept TDM guided dose adaptation.^110^ Gatti *et al*. demonstrated the successful implementation of TDM in the ICU. After an initial organizational phase that involved the formation of an expert clinical pharmacology advisory (ECPA) group including clinical pharmacologists, engineers, ICU clinicians and bioanalytical experts that oversaw various steps involved in TDM such as developing dosing algorithms, EHR integration and assay turnaround time.^111^ In the second phase, they assessed the impact of the ECPA on dose adaptation and turnaround times. They improved clinician acceptability by daily briefings in the ICU and the program led to improved service delivery, reduction in turnaround times and more dose adaptations.^111^ This example shows that feasibility and implementation studies involving key providers and stakeholders (representatives from clinical, microbiology, pharmacy, nursing, laboratory, information technology and hospital administrative services) are still required for the optimized use of TDM within a clinical environment.^107,111,112^ Stakeholder engagement should continue throughout the pre-implementation, implementation and post-implementation phases and should play an important role in iterative changes to a TDM service roll-out.^84,113^

## Future directions

Innovative developments designed to improve the clinical application of TDM are anticipated in the near future.^81^ Research on the development of mechanism-based models (that provide information on bacterial kill kinetics in the presence of varying antibiotic concentrations) that can be integrated with population PK to optimize antibiotic exposure is underway.^114^ Further research into improving existing population PK models using model averaging and continuous learning approaches is also required to improve clinical decision making and precision in dosing. The role of MIC-independent PK/PD matrices is also being studied to overcome the limitations associated with MIC measurement.^115^ Alternate sample types for TDM such as saliva, dried blood spots, ultrafiltrate from renal replacement therapy and interstitial fluid are being studied.^81,116,117^ Other promising technology that may revolutionize the way TDM is performed, interpreted and acted on is the field of wearable medical devices such as biosensors.^118^ A biosensor consists of an analytical device that converts biological signals (microbial DNA, antibiotics, enzymes) into measurable electrical signals.^118^ In a proof of concept, first in human study (*n* = 10), Rawson *et al.* used microneedle biosensors to measure phenoxymethylpenicillin concentrations in healthy volunteers and demonstrated good correlation between concentrations measured by microdialysis and plasma.^118^ Research using biosensor-based TDM in patients, if successful, will make real-time measurements of beta-lactam concentrations possible.^118^ Closed loop antibiotic delivery systems may also enable real-time dose adaptation in response to biosensor measured concentrations.^118^ The integration of newer technologies with electronic medical records will improve user-friendliness and could lead to the development of large databases with inputs that include a multitude of co-variates and clinical outcomes amenable to learning analytics.^119^ Advances in biosensor, MIPD, point of care technologies and cloud connectivity will lend themselves to wider application, accessibility and remote monitoring.^114^ These advances will assist in removing the requirement for large machines and complex sample processing techniques, making precision antibiotic dosing accessible to a variety of settings.^118^

## Conclusions

Beta-lactam TDM is a step toward precision medicine in the management of infection syndromes. The main role for beta-lactam TDM currently, is its utility in achieving and maintaining efficacy targets and avoiding toxic concentrations. Well-designed, prospective studies should include clearly defined populations with defined clinical, biochemical or bacteriological end points. The effectiveness of MIPD and software-guided dose optimization strategies should also be included in these studies. Recent literature on association of beta-lactam TDM and improved treatment success is encouraging. The lack of robust prospective data from RCTs on improved outcomes should not preclude the implementation of beta-lactam TDM. An important consideration is the significant requirements of infrastructure, personnel and expertise. Prospective studies demonstrating cost-effectiveness, feasibility and acceptability of TDM to healthcare providers will be required to justify the establishment of this intervention within the critical care setting.
